# Soybean Milk Inhibits Absorption and Intestinal Transmembrane Transport of Gegen in Rats

**DOI:** 10.1155/2017/7146813

**Published:** 2017-08-30

**Authors:** Xiao Ling, Yuqiang Xiang, Qingfa Tang, Zhen Jin, Feilong Chen, Xiaomei Tan

**Affiliations:** ^1^School of Traditional Chinese Medicine, Southern Medical University, Guangzhou 510515, China; ^2^Guangdong Provincial Key Laboratory of Chinese Medicine Pharmaceutics, Southern Medical University, Guangzhou 510515, China

## Abstract

Puerariae Lobatae Radix, known as Gegen in Chinese, is widely used to treat cardiovascular diseases, diabetes, and many other chronic illnesses. Flavonoids are the main active components in Gegen and are found in high concentrations in soybeans. Few studies, however, have focused on the effects of flavonoid-rich food on the absorption of Gegen. Here, we report an in vivo pharmacokinetic study on rats to explore the effects of soybean milk on the absorption of Gegen and an in vitro Ussing chamber study of puerarin intestinal transmembrane absorption. Area under the plasma concentration-time curve (AUC_0–*t*_) and maximum plasma concentration (*C*_max_) values of puerarin in rats were significantly decreased after drinking soybean milk, when taking Gegen decoction or a Gegen patent medicine (*P *< 0.01). In the Ussing chamber experiment, cumulative transmission (Qtn) after 2 h and apparent permeability coefficient (*P*app) were lower in the puerarin-daidzin and puerarin-soybean milk solution groups than in the puerarin group. Daidzin in soybean milk inhibited the transmembrane transport of puerarin, resulting in decreased bioavailability of puerarin in Gegen. The results of this study strongly suggest that Gegen should not be taken with flavonoid-rich food, particularly soybean products.

## 1. Introduction

Numerous studies have shown that simultaneous administration with food either promotes or inhibits drug absorption, altering pharmacokinetics (PK) and/or pharmacodynamics of drugs [1–6]. Numerous potentially clinically significant food-drug interactions are recognized by worldwide regulatory agencies, each with specific guidelines. The United States Food and Drug Administration (FDA) issued a guideline in 2002 stating recommendations for the design and methodology of studies on food-drug interactions [7]. These studies are critical in evaluating the appropriate dosing, timing, and formulation of new western drug candidates. However, as far as we know, scientific research on food-Chinese herb interactions has not been reported, especially regarding interactions with common foods. Chemical constituents of traditional Chinese medicines (TCMs) are complex. Many of these compounds are also present in food eaten daily. One of the mechanisms underlying food-drug interactions is that compounds in food may influence drug absorption via changes to intestinal biochemical processes and transporter-mediated efflux and uptake [8]. Compounds with similar structures often share the same transport mechanisms [9–12] and may influence the transport and absorption of each other. Therefore, the impact of food on TCMs needs to be studied more closely to provide guidelines on how to effectively administer TCMs.

Gegen, the root of* P. lobata* (Willd.) Ohwi, has been used as a food source, fodder, and medicine for thousands of years [13]. In China, numerous pharmaceutical dosage forms of Gegen are commercially available including tablets, pills, granules, and powders. Gegen is often used to treat cardiovascular diseases, cerebrovascular disorders, cancer, Parkinson's disease, Alzheimer's disease, diabetes, diabetic complications, and many other chronic diseases [14–17] that require daily medication. The representative functional compound of Gegen is puerarin, which has a similar structure to isoflavones found in soybeans. As one of the most common soybean products in East Asia, soybean milk is a standard component of Chinese breakfast and popular food in the greater Chinese region. Soybean milk is rich in isoflavanones such as daidzein and daidzin [18]. It has been indicated that daidzin may share active transporter proteins with puerarin. P-gp and MRP were reported as active transporters of puerarin in the Caco-2 cell monolayer model and daidzin is also a substrate of MRP2 [19–22]. Accordingly, daidzin in soybean milk may affect the absorption of puerarin by competing for transmembrane transport, which in turn affects the clinical efficacy of Gegen.

In this study, the effects of soybean milk on the absorption and intestinal transport of Gegen decoction and Gegen tablets (brand name: Yufeng Ningxin) [23] were studied using a PK in vivo rat study and an in vitro intestinal mucosal transport experiment with an Ussing chamber. A simple and rapid ultrahigh performance liquid chromatography-tandem mass spectrometry (UHPLC-MS/MS) method was developed to determine the concentration of puerarin in rat plasma and Ussing chamber samples. PK properties, Qtn (cumulative transmission), and* P*app (apparent permeability coefficient) of puerarin in different groups were analyzed to explain the influence of soybean milk on bioavailability and intestinal transport of Gegen in each formulation. To our knowledge, this is the first study to report the antagonistic effect of a common food on the absorption of a TCM, illustrating that interactions between TCMs and food should be carefully considered.

## 2. Experimental

### 2.1. Materials and Reagents

**Figure 1:**
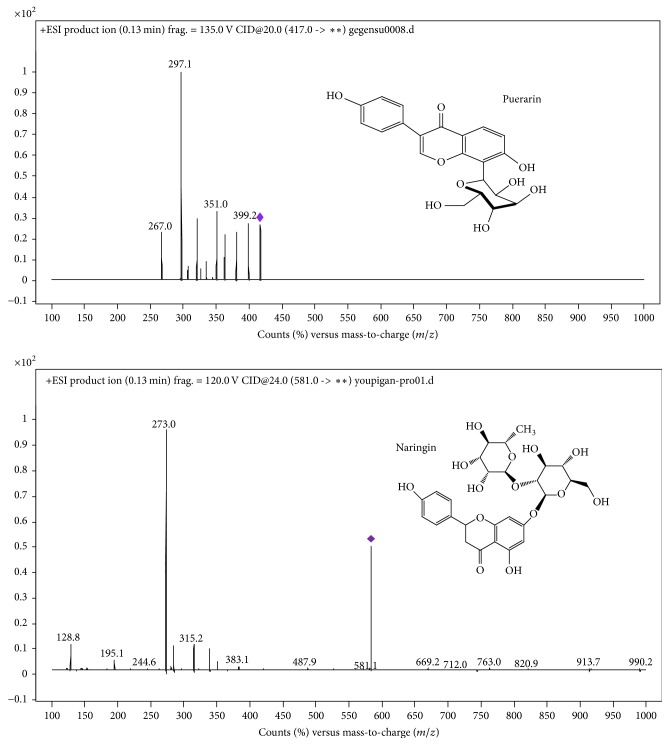
Product ion mass spectra and chemical structures of puerarin and naringin (IS). *∗∗* represents the molecular weight of the possible product ions.

Puerariae Lobatae Radix (*Pueraria lobata* (Willd.) Ohwi root, Chinese name: Gegen) was purchased from Zhixin Pharmaceutical Co., Ltd. (Guangzhou, China, batch number: 20140201) and was identified by Professor Liu Chuanming from the Traditional Chinese Medicine Identification Laboratory, Southern Medical University. Reference standards of puerarin (95.5% pure), daidzin (95.4% pure), and naringin (internal standard, 99% pure) were purchased from the National Institute for Food and Drug Control (Beijing, China). Chemical structures of compounds are shown in Figure 1. Yufeng Ningxin tablets were produced by Beijing Tongrentang Co., Ltd. (Beijing, China, batch number: 14120209). HPLC-grade acetonitrile and methanol were obtained from Merck (KGaA, Darmstadt, Germany).

Krebs-Ringer solution (0.37 mg/mL CaCl_2_·2H_2_O, 0.35 mg/mL KCl, 0.24 mg/mL MgCl_2_·6H_2_O, 0.19 mg/mL NaH_2_PO_4_·2H_2_O, 6.84 mg/mL NaCl, 2.10 mg/mL NaHCO_3_, and 1.98 mg/mL glucose) was prepared before each experiment. All reagents except acetonitrile and methanol were commercially available at analytical purity.

### 2.2. Preparation of Soybean Milk and Gegen Decoction

Soybean milk was prepared using a soybean milk machine (DJ12B-DSJ2, GD Midea Holding Co., Ltd., Guangdong, China). Soybean (100 g) and water (1000 mL) were added to the soybean milk machine and soybean milk was produced according to the automatic program of the machine.

Gegen decoction was prepared according to the Chinese Pharmacopoeia recommended dose. Gegen (15 g) was soaked in 200 mL of water for 30 min and kept boiling for 30 min. The boiling liquid was transferred to a container and another 200 mL of cold fresh water was added, before boiling the mixture for an additional 20 min. The liquids were combined to produce the final decoction, which was mixed and freeze-dried into powder. The method of puerarin content determination in the Gegen decoction is given in the Supplementary Materials available online at https://doi.org/10.1155/2017/7146813.

### 2.3. Animal Grouping for the PK Study

Specific pathogen-free male Sprague-Dawley rats (SD rats), weighing 200–250 g, were provided by the Experimental Animal Center, Southern Medical University (Guangzhou, China), and were acclimatized to the lab for 3 days before commencing the experiments. The rats were provided with standard chow and water ad libitum and maintained under controlled conditions (temperature: 25 ± 1°C, relative humidity: 65 ± 10%, and a 12 h light/dark cycle with lights on at 7:00 a.m.). All studies on animals were conducted in accordance with the guidelines of the Committee on the Care and Use of Laboratory Animals in China.

SD rats (24) were divided into Gegen decoction group (G group), Gegen decoction-soybean milk solution group (GS group), Yufeng Ningxin group (Y group), and Yufeng Ningxin-soybean milk group (YS group).

### 2.4. Pharmacokinetic Studies

Rats from G, GS, Y, and YS groups were orally given one dose of Gegen decoction solution, Gegen decoction-soybean milk solution, Yufeng Ningxin aqueous solution, and Yufeng Ningxin-soybean milk solution, respectively. The doses for each group were equivalent to 10 mg/kg puerarin. Plasma samples were collected from the orbital venous plexus at 0 (predose), 0.08, 0.15, 0.5, 1, 1.5, 2, 4, 6, 8, 10, 12, and 24 h after administration. The plasma was immediately separated via centrifugation at 5000 rpm for 5 min and stored at −20°C until analysis.

The validated HPLC/MS/MS method described below was applied to analyze puerarin in rat plasma of these four groups. Maximum plasma concentration (*C*_max_) and maximum plasma time (*T*_max_) were obtained from the observed data, whereas area under the plasma concentration-time curve (AUC_0–*t*_), clearance rate (CL*z*/*F*), mean resident time (MRT), elimination half-life (*t*_1/2_), and volume of distribution (*Vz*/*F*) were calculated using DAS software version 3.0 (Mathematical Pharmacology Professional Committee of China, Shanghai, China) with a noncompartmental method.

### 2.5. Intestinal Absorption Study

Rats were fasted for 12 h before the experiment, with water provided ad libitum. Rat jejuna were collected from the fasting rats under 10% chloral hydrate anesthesia. The jejuna were washed with physiological saline and dipped in ice-cold Krebs-Ringer solution with O_2_/CO_2_ (95/5, v/v). To obtain intestinal mucosa, the muscularis mucosae were carefully removed from the serosa side. Intestinal mucosa was immobilized on tissue plates and inserted into the perfusion pool (area: 1.78 cm^2^) of the Ussing chamber. Freshly prepared Krebs-Ringer solution (5 mL), saturated with mixed gas (95% O_2_ and 5% CO_2_) and preheated to 38°C, was added to both sides of the perfusion pool.

Six different drug combination groups (Y group, YS group, puerarin group (P group), puerarin-daidzin group (PD group), puerarin-verapamil group (PV group), and puerarin and soybean milk group (PS group)) were designed to study the effect of soybean milk on intestinal transport of puerarin. Drugs were administered in the following doses: 5.77 mg/mL Yufeng Ningxin (equal to 0.3 mg/mL puerarin) for Y group, 5.77 mg/mL Yufeng Ningxin soybean milk solution for YS group, 0.3 mg/mL puerarin for P group, 0.3 mg/mL puerarin and 0.1 mg/mL daidzin for PD group, 0.3 mg/mL puerarin and 0.2 mg/mL verapamil for PV group, and 0.3 mg/mL puerarin-soybean milk solution for PS group. Once the drug was added to the perfusion pool, 200 *μ*L of liquid was collected after 0.5, 1, 1.5, and 2 h on the serosa side. This was repeated for each treatment tested, and the same volume (200 *μ*L) of Krebs-Ringer solution was added to the sample side after each sampling. To maintain intestinal activity, a mixture of gases (95% O_2_ and 5% CO_2_) was applied during the experiment and the temperature of each perfusion pool was maintained at 38°C. Voltage and current were recorded to help evaluate intestinal activity, which was considered poor if voltage and current changed sharply. The Ussing chamber samples were frozen at −20°C before the test.

### 2.6. Sample Preparation

IS (naringin) solution (20 *μ*L, 4 *μ*g/mL) was added to 100 *μ*L of plasma. After vortex-mixing for 30 s, 800 *μ*L of methanol was added. The mixture was vortexed for 3 min and centrifuged at 15000 rpm for 10 min at 4°C. The supernatant was transferred into an Eppendorf tube and dried under nitrogen. The dried residue was dissolved with 200 *μ*L methanol and centrifuged at 15000 rpm for 10 min. The supernatant was transferred into autosampler vials, and 2 *μ*L was injected into the UHPLC/MS/MS system for analysis.

The Ussing chamber samples were prepared as follows: IS solution (20 *μ*L, 10 *μ*g/mL) was added to 100 *μ*L of each sample from the Ussing chamber. The samples were dried under nitrogen at 25°C. Dried residues were dissolved with 200 *μ*L methanol and centrifuged at 15000 rpm for 10 min. The supernatant was transferred into autosampler vials, and 2 *μ*L was injected into the UHPLC/MS/MS system for analysis.

### 2.7. UHPLC/MS/MS Analysis

The samples were analyzed using a 6410 triple-quad UHPLC-MS system (Agilent Technologies Inc., CA, USA) with a C18 column (100 nm × 2.5 mm, 3.5 *μ*m, Agilent Technologies Inc.). Multiple reaction monitoring (MRM) mode was chosen to quantify the investigated analytes. The parameters are shown in Table 1. The product ion mass spectra of puerarin and naringin are shown in Figure 1. The TQ mass spectrometer was operated with a capillary voltage of 3 kV and an ion source temperature of 350°C. Electrospray ionization source was used in positive ionization mode.

HPLC conditions included the mobile phase of A (0.1% aqueous formic acid) and B (acetonitrile), gradient elution of 15–30% B for 0–2.5 min, 30–35% B for 2.5–3.5 min, and 35–15% B for 3.5–5 min, flow rate of 0.4 mL/min, and sample injection volume of 2 *μ*L.

### 2.8. Preparation of Quality Control Samples

#### 2.8.1. Preparation of Quality Control (QC) for Plasma Sample Analysis

Standard stock solution of puerarin was diluted with methanol to obtain a concentration of 1 mg/mL and further diluted through a series of working solutions to the desired concentration of 10 ng/mL. A working solution of naringin (4 *μ*g/mL) was also prepared with methanol.

Aliquots (100 *μ*L) of the working solutions were spiked with blank rat plasma to obtain calibration standards and QC samples. Final concentrations of calibration standard samples for puerarin were 5–1000 ng/mL. QC samples of puerarin were prepared at concentrations of 15, 100, and 750 ng/mL. All solutions were stored at 4°C before use, whereas QC samples were stored at −20°C.

#### 2.8.2. Preparation of QC for Ussing Chamber Samples

Standard stock solutions were prepared as described in Section 2.8.1. A series of working solutions of puerarin and naringin were diluted with methanol to reach final concentrations of 35 *μ*g/mL and 10 *μ*g/mL, respectively. Aliquots (100 *μ*L) of the working solutions were spiked with Krebs-Ringer solution to obtain calibration standards and QC samples. Final concentrations of calibration standard samples for puerarin were 5–3500 ng/mL. Puerarin QC samples were prepared at concentrations of 20, 1000, and 2500 ng/mL. All solutions were stored at 4°C before use, whereas QC samples were stored at −20°C.

### 2.9. Method Validation for Plasma and Ussing Chamber Samples

Because of the differences in the biological matrix of plasma samples and intestinal absorption samples, the methods for these two types of samples were validated for selectivity, linearity, precision, accuracy, extraction recovery rates, matrix effects, and stability during sample storage and processing procedures according to the FDA guidelines for the validation of bioanalytical methods [24]. Validation methods for the Ussing chamber samples were the same as for the plasma samples except that the blank plasma was replaced by Krebs-Ringer solution in the intestinal absorption study.

Specificity was assessed by analyzing blank plasma, blank plasma spiked with puerarin, and real plasma samples from rats after oral administration of drugs.

Linearity was evaluated by preparing 10 different concentrations of samples in plasma. Calibration curves were prepared using the standard plasma samples described above and constructed from peak area ratios of puerarin to IS versus the plasma concentrations using a 1/*x*^2^ weighted linear least-squares regression model.

Within- and between-batch precision and accuracy were investigated by determining QC samples at three different concentrations. Interday assay accuracy and precision were established by performing calibration tests for 3 consecutive days. Accuracy was required to be within 85–115% and the precision error not to exceed 15%.

Extraction recovery of puerarin from rat plasma was determined at three concentrations of plasma QC samples and calculated as the ratio of analyte peak area from extracted plasma QC samples to that from extracted blank plasma spiked with standard working solutions. The matrix effect was investigated by comparing the peak area of the analytes added to the preextracted plasma from untreated rats with that of the analytes dissolved in a matrix component-free reconstitution solvent.

The stability of the analytes in the plasma was assessed using three QC plasma samples stored under four different conditions. One set of samples (postpreparation) was stored for 12 h at room temperature, one set was stored for 30 days at −20°C, one set (prepreparation) was stored for 12 h at 20°C, and one set was exposed to three freeze-thaw cycles (−20°C to room temperature).

### 2.10. Data Analysis

Pharmacokinetic parameters (AUC, MRT, *T*_1/2*z*_, *T*_max_, *V*_*z*_/*F*, and Cl_z_/*F*) were calculated by noncompartmental analysis of the plasma concentration versus time data using the Drug and Statistics (DAS 3.0) software (Bio Guider Co., Shanghai, China). Qtn and* P*app of puerarin in different groups in Ussing chamber experiment were calculated according to the following formula [25]:

(1) $$\begin{array}{c} Qtn=5Ctn+\overset{n-1}{\underset{i=1}{\sum }}0.2Ct\left(\;n-1\;\right), \end{array}$$

where 0.2 and 5 indicate that the sampling volume and the volume added to the solution were 0.2 mL and 5 mL, respectively, and Ctn indicates the drug concentration in the receiving room at a given time point. The apparent coefficient of permeability was determined as follows:

(2) $$\begin{array}{c} Papp=\left(\;\frac{dQ}{dt}\;\right)\times \left(\;\frac{1}{AC_{0}}\;\right), \end{array}$$

where *dQ*/*dt* represents the slope of the linear regression for time-cumulative transmission, *A* represents effective permeability area (1.78 cm^2^ in this study), and *C*_0_ represents initial drug concentration in the diffusion chamber.

All PK parameters were analyzed using SPSS 13.0 (Statistical Package for the Social Sciences, SPSS Inc., Chicago, USA).* P* < 0.05 was considered statistically significant.

## 3. Results

### 3.1. PK Studies of Gegen

**Figure 2:**
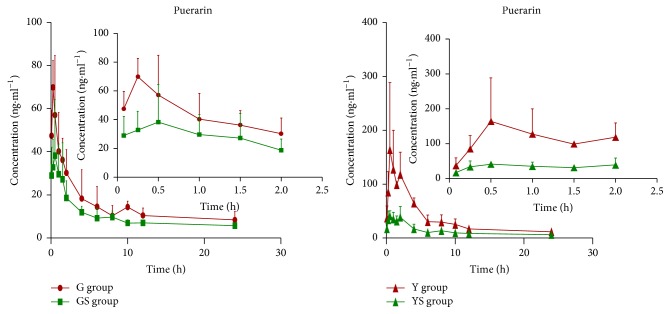
Mean concentration-time profiles of puerarin in rat plasma after oral administration of Yufeng Ningxin tablets and Gegen decoction (equivalent to 10 mg/kg puerarin). Each pointer presents the mean ± SD (*n* = 6).

The shape of the concentration-time curve of puerarin was similar in G, GS, Y, and YS groups (Figure 2). Puerarin plasma concentration increased rapidly and reached a peak 15 to 30 min after administration, decreasing rapidly 1 h after administration and remaining quite low after 10 h. Puerarin plasma concentrations were significantly higher in G group than in GS group rats at most time points. Similarly, AUC_0–*t*_ and *C*_max_ values of the GS group animals were significantly lower than those of G group animals (*P *< 0.01) (Table 2). Clearance of puerarin in rats was accelerated after drinking soybean milk, with MRT and* t*_1/2_ decreasing and* Vz/F* and CL_*z*_/*F* values of puerarin increasing.

Puerarin plasma concentrations were significantly higher in Y than in YS group rats at most time points. This difference was also reflected in values of PK parameters. AUC_0–*t*_ and *C*_max_ values of Y group animals were 2.35 and 3.69 times higher than those of YS group animals, respectively (*P* < 0.01). MRT and* t*_1/2_ decreased, whereas *Vz*/*F* and CL_*z*_/*F* values of puerarin increased after drinking soybean milk (Table 3).

### 3.2. Absorption Study Using the Ussing Chamber

**Figure 3:**
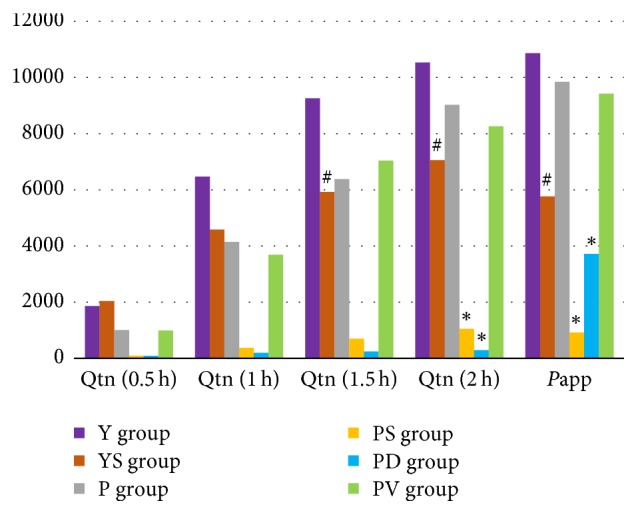
Qtn and* P*app of puerarin in the intestinal absorption study in six groups with the same puerarin concentration (0.3 mg/mL). Y group, YS group, P group (puerarin aqueous solution), PS group (puerarin-soybean milk solution), PD group (puerarin and daidzin), and PV group (puerarin and verapamil). ^#^*P* < 0.01 compared with Y group; ^*∗*^*P* < 0.01 compared with P group.

In this experiment, the effects of daidzin and soybean milk on the intestinal absorption of puerarin were explored through intestinal absorption studies of different combinations of soybean glycosides, soybean milk, and puerarin. The results are shown in Figure 3.

*P*app and Qtn of puerarin in YS group were remarkably lower compared to Y group (*P*app: 5764.33 versus 10863.76 cm/s; Qtn after 1.5 h: 5921.57 versus 9258.91 ng/mL; Qtn after 2 h: 7052.60 versus 10528.72 ng/mL, resp.).* P*app and Qtn of puerarin showed a significant difference (*P* < 0.01) between P and PD groups. Qtn after 2 h was lower in the PD group than in the P group (5157.06 versus 9025.67 ng/mL), comparable to* P*app (5996.97 versus 9840.53 cm/s).

The same phenomenon was found in PS group. Qtn and* P*app for puerarin in PS group were 5268.42 ng/mL and 5894.89 cm/s, respectively, a significant reduction compared to P group (*P *< 0.05). However, no significant differences in* P*app and Qtn of puerarin were observed between P and PV groups.

### 3.3. UHPLC/MS/MS Analytical Method Validation for Plasma Samples

#### 3.3.1. Specificity

**Figure 4:**
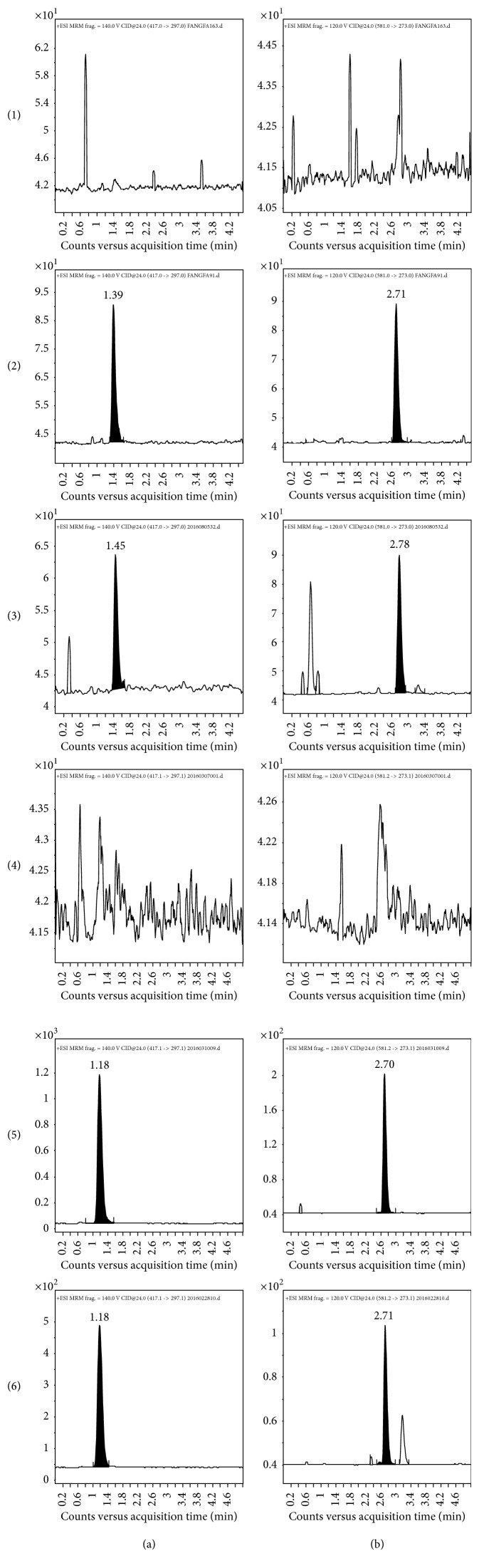
Representative multiple reaction monitoring chromatograms of (a) puerarin and (b) naringin (IS) in (1) blank plasma, (2) blank plasma spiked with standard solution and internal standard, (3) plasma sample after oral administration of puerarin, (4) blank Krebs-Ringer solution, (5) blank Krebs-Ringer solution spiked with standard solution and internal standard, and (6) Ussing chamber sample.

As no endogenous substances were found to interfere with the analytes or the IS in the plasma, the method exhibited good specificity. Typical MRM chromatograms are shown in Figure 4.

#### 3.3.2. Linearity of Calibration Curves and Lower Limit of Quantification (LLOQ)

Linear regression equation and correlation coefficient of puerarin in plasma were* y* = 80.617*x* − 1.204 and* r*^*2*^ = 0.996, whereas linear ranges and LLOQ were 5–1000 ng/mL and 5.0 ng/mL, respectively.

#### 3.3.3. Accuracy, Precision, Recovery, and Matrix Effect

Intra- and interday accuracy and precision of puerarin determination in plasma were satisfactory (detailed results are shown in Table 4). Extraction recoveries and the matrix effect of puerarin in plasma are shown in Table 4. Extraction recoveries of QC samples in plasma varied from 98.50% to 114.85%. Average matrix effects were found to be within the acceptable range (87.85–97.49%).

#### 3.3.4. Stability

Puerarin was found to be stable in plasma under four different storage conditions analyzed. Ranges of precision and accuracy of the method were 1.04–14.24% and −14.09 to 14.02%, respectively (Table 5).

### 3.4. UHPLC-MS/MS Analytical Method Validation for Ussing Chamber Samples

#### 3.4.1. Specificity

As no endogenous substances were found to interfere with the analytes or the IS in the plasma, the method exhibited good specificity. Typical MRM chromatograms are shown in Figure 3.

#### 3.4.2. Linearity of Calibration Curves and LLOQ

Linear regression equation and correlation coefficient of puerarin in Ussing chamber samples were* y* = 0.0072*x* + 0.1044 and* r*^2^ = 0.998, respectively. Linear ranges and LLOQ were 5–3500 ng/mL and 5.0 ng/mL, respectively.

#### 3.4.3. Accuracy, Precision, Recovery, and Matrix Effect

Detailed results for intra- and interday accuracy and precision of determining puerarin content in Ussing chamber samples are shown in Table 6. Extraction recovery of Ussing chamber QC samples varied from 87.60% to 97.40%. The average matrix effects were from 98.88% to 109.32% (Table 6).

#### 3.4.4. Stability

Analytes in Ussing chamber samples were stable under the four test conditions. Precision and accuracy ranges for puerarin were −2.32 to 9.20% and −6.40% to 7.20%, respectively (Table 7).

These results demonstrate that this bioanalytical method meets FDA validation guidelines.

## 4. Discussion

The validated analysis method was successfully applied to PK study of puerarin in rat plasma after oral administration of Gegen decoction and Yufeng Ningxin tablets. Selectivity, linearity, precision, accuracy, extraction recovery rates, matrix effects, and stability during sample storage and processing procedures all met FDA guidelines [20].

According to results of the PK study, soybean milk significantly reduced AUC and *C*_max_ while increasing *Vz*/*F* and CL*z*/*F*. Significant reduction in AUC_0–*t*_ and *C*_max_ suggests greatly decreased puerarin concentration. Many pharmacological effects of Gegen, especially cardiovascular and antidiabetic effects, are closely associated with puerarin, which is its main active ingredient. Low absorption of puerarin would negatively affect the efficacy of Gegen. Furthermore, soybean milk also increased *Vz*/*F* and CLz/*F*. Increased *Vz*/*F* and CL*z*/*F* in the presence of soybean milk indicates that puerarin was widely distributed in the body and cleared faster from the blood, respectively. Taken together, these data imply that larger and more frequent doses of Gegen are required to maintain effective drug plasma concentration in patients concurrently consuming soybean milk. Furthermore, similar shapes of the concentration-time curves and *T*_max_ (0.46 versus 0.79 h in G and GS group, resp.; 1.08 versus 0.88 h in Y and YS groups, resp.) in the four groups indicate that gastrointestinal absorption site of puerarin and gastrointestinal motility rate were not affected by soybean milk in this study.

The Ussing chamber results supported this hypothesis. As shown in Figure 4, based on decreasing Qtn and* P*app in YS and PS groups, the amount of puerarin that passed through the intestinal mucosa decreased remarkably after soybean milk treatment. Similar results in PD group indicated that daidzin also inhibited the transmembrane absorption of puerarin. These results suggest that soybean milk reduced the absorption of puerarin by inhibiting transmembrane absorption and that daidzin may be the main mediator of the effect.

However, mechanisms behind daidzin-mediated inhibition of transmembrane absorption of puerarin remain unknown. As competitive inhibition does not affect passive diffusion, absorption differences observed in this study should originate from the active transport processes. Active transport of puerarin was shown to be mediated by P-gp and MRP transporters [16–18]. However, Ussing chamber results in this study indicate that daidzin-mediated inhibition of intestinal transmembrane transport of puerarin does not depend on P-gp and MRP. As these transporters are involved in drug efflux from cells, their inhibition by daidzin is expected to reduce the discharge of puerarin and increase its concentration. However, puerarin Qtn and* P*app in PD group were lower than in P group (5157.06 versus 9025.67 ng/mL, 5996.97 versus 9840.53 cm/s), which does not support this hypothesis. Although at least one study has reported puerarin as a possible P-gp substrate [18], this inference could not be verified in our study. No significant differences were observed in Qtn and* P*app values between P and PV groups. These results suggest that the absorption differences are unrelated to P-gp. The differences may be related to other transporters, however, and further studies are needed to elucidate the specific proteins involved.

## 5. Conclusion

In this study, we analyzed the interaction between soybean milk and Gegen using the Ussing chamber and PK experiments. Our results indicate that soybean milk reduces absorption and accelerates clearance of puerarin from Gegen due to soybean milk isoflavonoids-mediated inhibition of puerarin transport and suggest that, in order to ensure the clinical effects of puerarin, the use of soybean preparations should be avoided during treatment.

## Supplementary Material

Puerarin content determination in Yufeng Ningxin Tablets and Gegen decoction and daidzin content determination in Soybean milk.

## Figures and Tables

**Table 1:** MRM parameters of puerarin, daidzin, and naringin.

Compound	Precursor ion	Product ion	Dwell	Fragment	Collison energy	Polarity
Puerarin	417.0	297.0	200	135.0	20.0	+
Daidzin	417.0	255.2	200	135.0	10.0	+
Naringin	581.0	273.0	200	120.0	24.0	+

**Table 2:** Pharmacokinetic parameters of puerarin after oral administration of Gegen decoction to rats (*n* = 6, $\overset{-}{\chi }\pm SD$).

	G group	GS group
AUC_0–*t*_ (ng·mL^−1^ h)	346.34 ± 49.91	238.57 ± 48.53^*∗∗*^
MRT_0–*t*_ (h)	9.90 ± 0.88	8.47 ± 0.76
*t*_1/2_ (h)	24.99 ± 18.65	16.46 ± 9.68^*∗∗*^
*T*_max_ (h)	0.46 ± 0.29	0.79 ± 0.46
*C*_max_ (ng·mL^−1^)	76.55 ± 14.69	52.35 ± 15.73^*∗∗*^
*V*_*z*_/*F* (L·kg^−1^)	533.85 ± 273.81	724.71 ± 357.63^*∗∗*^
CL_*z*_/*F* (L·kg^−1^)	19.75 ± 8.47	28.19 ± 13.36^*∗∗*^

^*∗∗*^ *P* < 0.01 compared with G group; G group was given Gegen decoction; GS group was given Gegen decoction soybean milk solution.

**Table 3:** Pharmacokinetic parameters of puerarin after oral administration of Yufeng Ningxin tablets to rats (*n* = 6, $\overset{-}{\chi }\pm SD$).

	Y group	YS group
AUC_0–*t*_ (ng·mL^−1^ h)	730.46 ± 150.63	246.69 ± 106.00^##^
MRT_0–*t*_ (h)	4.95 ± 1.33	4.64 ± 1.34
*t*_1/2_ (h)	9.54 ± 4.21	8.58 ± 10.10
*T*_max_ (h)	1.08 ± 0.58	0.88 ± 0.63
*C*_max_ (ng·mL^−1^)	188.21 ± 118.78	51.80 ± 17.67^##^
*V*_*z*_/*F* (L·kg^−1^)	150.83 ± 64.98	312.97 ± 137.45^##^
CL_*z*_/*F* (L·kg^−1^)	11.17 ± 1.84	37.15 ± 14.10^##^

^##^ *P* < 0.01 compared with Y group; Y group was given Yufeng Ningxin aqueous solution; YS group was given Yufeng Ningxin soybean milk solution. All the four dose regimens are equal to 10 mg/kg puerarin.

**Table 4:** Summary of recovery, matrix effect, accuracy, and precision of puerarin for the UHPLC-MS/MS method in rat plasma (*n* = 6, $\;\overset{-}{\chi }\pm SD$).

		Spiked conc. (ng·mL^−1^) of puerarin		
		15	100	750
Recovery (%)		98.50 ± 14.51	111.05 ± 15.06	114.85 ± 6.06
Matrix effect (%)		96.50 ± 12.28	97.49 ± 6.42	87.85 ± 9.66
Precision	Intraday	14.73	13.56	7.68
RSD%	Interday	8.99	8.71	4.63
Accuracy	Intraday	−5.04	11.05	1.02
RE%	Interday	−3.02	7.54	11.11

**Table 5:** Summary of stability of puerarin for the UHPLC-MS/MS method in rat plasma (*n* = 6).

		Spiked conc. (ng·mL^−1^) of puerarin		
		15	100	750
3 freeze-thaw cycles	Precision (RSD%)	14.20	2.84	6.24
	Accuracy (RE%)	−12.22	11.02	13.41
Long-term (28 days, −20°C)	Precision (RSD%)	8.97	5.71	5.06
	Accuracy (RE%)	−14.09	−0.56	10.58
Postpreparation (12 h, 20°C)	Precision (RSD%)	14.24	8.12	3.59
	Accuracy (RE%)	14.02	−3.19	11.79
Prepreparation (12 h, 20°C)	Precision (RSD%)	13.03	1.04	7.29
	Accuracy (RE%)	−10.93	2.42	5.95

**Table 6:** Summary of recovery, matrix effect, accuracy, and precision of puerarin for the UHPLC-MS/MS method in Ussing chamber samples (*n* = 6, $\overset{-}{\chi }\pm SD$).

		Spiked conc. (ng·mL^−1^) of puerarin		
		20	1000	2500
Recovery (%)		97.40 ± 0.80	92.00 ± 5.80	87.60 ± 4.20
Matrix effect (%)		109.32 ± 4.18	100.21 ± 2.02	98.88 ± 2.16
Precision	Intraday	7.51	3.56	6.13
RSD%	Interday	5.82	5.23	6.12
Accuracy	Intraday	1.15	5.00	−5.60
RE%	Interday	−4.25	7.00	−6.40

**Table 7:** Summary of stability of puerarin for the UHPLC-MS/MS method in Ussing chamber samples (*n* = 6).

		Spiked conc. (ng·mL^−1^) of puerarin		
		20	1000	2500
3 freeze-thaw cycles	Precision (RSD%)	8.36	3.33	9.20
	Accuracy (RE%)	2.39	7.20	0.09
Prepreparation (24 h, 4°C)	Precision (RSD%)	5.80	5.21	6.14
	Accuracy (RE%)	−4.25	7.00	−6.40
Postpreparation (12 h, 20°C)	Precision (RSD%)	6.66	8.32	1.25
	Accuracy (RE%)	−2.38	−5.38	−6.1
Prepreparation (12 h, 20°C)	Precision (RSD%)	7.23	2.13	−2.32
	Accuracy (RE%)	4.29	3.19	−1.73
