# Supplementary material for: Soybean Milk Inhibits Absorption and Intestinal Transmembrane Transport of Gegen in Rats
Source: Evid Based Complement Alternat Med. 2017 Aug 30;2017:7146813. doi: 10.1155/2017/7146813 (PMC5602493; doi:10.1155/2017/7146813)
Supplement: Supplementary file 1 — Puerarin content determination in Yufeng Ningxin Tablets and Gegen decoction and daidzin content determination in Soybean milk. [file 7146813.f1.doc]

**1.Content determination of Puerarin in Yufeng Ningxin Tablets and Gegen decoction.**

1.1Materials and reagents

Reference Standards of Puerarin（purity was 95.5%）was purchased from the National Institute for Food and Drug Control (Beijing, China). Yufeng Ningxin tablets were produced by Beijing Tongrentang co., ltd. (Beijing, China). Acetonitrile and methanol of HPLC grade were obtained from Merck (KGaA, Darmstadt, Germany).C18 column（4.6 x 250mm，Nacalai Tesque，Inc. Kyoto，Japan）

1.2. HPLC analytical method^1^

The method is recorded in Chinese Pharmacopoeia. The mobile phase was composed of methanol and water using isocratic elution of 25%A with a flow rate set at 1 mL min^-1^. Detection wavelength was 250nm and injection volume was 10μL.

1.3. Sample preparation^1^

Preparation of standard solution：The standard solution was dissolved in 30% ethanol to obtain a concentration of 80μg/mL for Puerarin. The inject volume was 10μL.

Preparation of product solution(Yufeng Ningxin tablets)：10 tablets of Yufeng Ningxin（each weighted 0.25g） were used after being removed the coating. The tablets were grinded into flour and 50mg powder were transferred in a conical flask with plug together with 50mL 30% ethanol. The whole conical flask was weighted before ultrasonic for 20min（250W, frequency 33kHz. It was weighted again and the reduced weight was supplemented with 30% ethanol. The solution was filtrated to obtain filtrate for analysis. The inject volume was 10μL.

Preparation of product solution (Gegen decoction):100mg Gegen decoction powders were transferred in a conical flask with plug together with 50mL 30% ethanol. The whole conical flask was weighted before ultrasonic for 20min（250W, frequency 33kHz. It was weighted again and the reduced weight was supplemented with 30% ethanol. The solution was filtrated to obtain filtrate for analysis. The inject volume was 10μL.

1.4 Method validation for Gegen decoction

The method described herein was validated for linearity, precision, repeatability, recovery rates. The linear relationship was evaluated by preparing six different concentrations of Puerarin. The linear regression equation and correlation coefficient of Puerarin was y = 48.064x + 112.84 R² = 0.9995. The linear ranges and LLOQ were 10-200 μg/ml and 10 μg/ml, respectively.
 Take the same test sample and detect 6 times according to the above condition in 1.2 for precision validation. Calculate the RSD of the peak area of Puerarin. Results showed that the RSD was 0.75%.

For the repeatability study, 6 batches of samples were prepared and detected according to the above condition in 1.2. Results showed that the RSD of the peak area of Puerarin was 0.87%.

Recovery rates: 50μL 400μg/mL Puerarin solution were added into 50μL Gegen decoction solution with known content of Puerarin. The mix samples were prepared and detected by the same method described in above 1.2 and 1.3 section. The result was shown in table 1.

Table1.The recoveries of Puerarin

| The content in Gegen decoction solution(μg) | Adding amount(μg) | Measured value(μg) | Recovery rates（%） | Mean recovery rates（%） | RSD  （%） |
| --- | --- | --- | --- | --- | --- |
| 193.58 | 200 | 400.03 | 101.64 |  |  |
| 194.60 | 200 | 381.89 | 96.78 |  |  |
| 192.59 | 200 | 401.15 | 102.18 | 99.90 | 2.35 |
| 191.19 | 200 | 381.21 | 97.45 |  |  |
| 192.99 | 200 | 399.87 | 101.75 |  |  |
| 193.58 | 200 | 392.08 | 99.62 |  |  |

1.4. Results

The Puerarin in each tablet of Yufeng Ningxin was 13.9mg. The Puerarin in Gegen decoction powders was 88.26mg/g.

Chromatogram of Yufeng Ningxin tablets ，Puerarin in Gegen decoction and Puerarin were show in Fig 1，Fig2 and Fig3.

Fig 1. HPLC Chromatogram of Yufeng Ningxin tablets

Fig 2. HPLC Chromatogram of Gegen decoction

Fig 3. HPLC Chromatogram of Puerarin

[1] Chinese Pharmacopoeia Commission. Pharmacopoeia of the people's Republic of China[M]. Part 1. Beijing. Chinese medicine science and Technology Press.2015, 1653

**2.Content determination of Daidzin in Soybean milk**

2.1. Materials and reagents

Reference Standards of Daidzin（purity was 95.4%）was purchased from the National Institute for Food and Drug Control (Beijing, China). Yufeng Ningxin tablets were produced by Beijing Tongrentang co., ltd. (Beijing, China). Acetonitrile and methanol of HPLC grade were obtained from Merck (KGaA, Darmstadt, Germany). C18 column（4.6 x 250mm，Nacalai Tesque，Inc. Kyoto，Japan）

2.2. HPLC analytical method

The mobile phase was composed of A (0.1% aqueous formic acid) and B (acetonitrile) using a gradient elution of 15% B for 0–7 min, 15-30% B for 7-17 min, 30%-40%B for 17-25min, 40%-15%B for 25-30min with a flow rate set at 1 mL min-1. The auto sampler was conditioned at 25°C and the sample injection volume was 5 μL. Detection wavelength was 270nm.

2.3. Sample preparation

Add 2.5mL cyclohexane into 10mL soybean milk. Discard the upper layer of fat after the ultrasonic extraction for 10min. 2mL of the remain was added 8mL methanol and then be extracted for another 10min by ultrasonic. The solution was centrifuged at 3000 rpm for 10 min. Supernatants were transferred and dried by water bath at 70℃. The residue was dissolved with 1mL methanol and then was filtered with 0.22μm membrane for HPLC analysis.

2.4. Method validation for soybean milk samles

The method described herein was validated for linearity, precision, repeatability, recovery rates. The linear relationship was evaluated by preparing six different concentrations of Daidzin. The linear regression equation and correlation coefficient of Daidzin was y = 0.0722x + 2.2812，r^2^=0.9998. The linear ranges and LLOQ were 10-500 μg/ml and 10 μg/ml, respectively.
 Take the same test sample and detect 6 times according to the above condition in 2.2 for precision validation. Calculate the RSD of the peak area of Daidzin. Results showed that the RSD was 1.25%.

For the repeatability study, 6 batches of samples were prepared and detected according to the above condition in 2.2. Results showed that the RSD of the peak area of Daidzin was 1.61%.

Recovery rates: 50μL 50μg/mL Daidzin solution were added into 50μL soybean milk with known content of Daidzin. The mix samples were prepared and detected by the same method described in above 2.2 and 2.3 section. The result was shown in table 2.

Table2.The recoveries of Daidzin

| The content in soybean milk(μg) | Adding amount(μg) | Measured value(μg) | Recovery rates（%） | Mean recovery rates（%） | RSD  （%） |
| --- | --- | --- | --- | --- | --- |
| 3.43 | 2.5 | 6.10 | 102.87 |  |  |
| 2.71 | 2.5 | 5.19 | 99.62 |  |  |
| 3.00 | 2.5 | 5.40 | 98.18 | 100.91 | 2.32 |
| 3.27 | 2.5 | 5.90 | 102.25 |  |  |
| 3.10 | 2.5 | 5.81 | 103.75 |  |  |
| 3.23 | 2.5 | 5.66 | 98.78 |  |  |

2.5 Results

The Daidzin in soybean milk was 3.12mg/mL.Chromatogram of soybean milk and Daidzin were show in Fig 4 and Fig5.

Fig4. HPLC Chromatogram of soybean milk

Fig 5. HPLC Chromatogram of Daidzin
